# Cough Burden and Sleep Quality in Patients with Idiopathic Pulmonary Fibrosis Receiving Antifibrotic Therapy: A Cross-Sectional Study in Türkiye

**DOI:** 10.3390/jcm15093521

**Published:** 2026-05-05

**Authors:** Halit Kazci, Utku Tapan, Ozge Oral Tapan

**Affiliations:** Department of Pulmonology, Faculty of Medicine, Mugla Sitki Kocman University, Mugla 48000, Türkiye; halitkazci@hotmail.com (H.K.); utkutapan@mu.edu.tr (U.T.)

**Keywords:** idiopathic pulmonary fibrosis, chronic cough, sleep quality, antifibrotic therapy, quality of life

## Abstract

**Background and Objectives:** Idiopathic pulmonary fibrosis (IPF) is frequently accompanied by chronic cough, which may negatively affect sleep quality. However, the relationship between cough burden and sleep disturbances in patients undergoing antifibrotic therapy remains inadequately defined. This study aimed to investigate the association between cough and sleep quality in patients with IPF and to evaluate the potential effects of antifibrotic treatment on these outcomes. **Materials and Methods:** This cross-sectional analytical study was conducted at a tertiary care center in Türkiye between January 2019 and December 2024. Patients with a diagnosis of IPF who were receiving antifibrotic therapy (nintedanib or pirfenidone) were consecutively recruited from the pulmonology outpatient clinic. Sleep quality was assessed using the Pittsburgh Sleep Quality Index (PSQI), and cough-related quality of life was evaluated with the Leicester Cough Questionnaire (LCQ). Pre-treatment data were obtained retrospectively based on patient recall, and follow-up assessments were conducted during ongoing therapy. Correlation analyses and multivariable linear regression models were used to identify factors associated with sleep quality. **Results:** The study included 74 patients, with a mean age of 68.6 ± 6.8 years. At baseline, 87.8% of patients had poor sleep quality (PSQI ≥ 5). During antifibrotic therapy, PSQI scores significantly improved (median 9 [IQR: 6–12] vs. 6 [IQR: 5–8], *p* < 0.001), accompanied by a clinically meaningful increase in LCQ total score (13.28 ± 2.86 vs. 16.06 ± 2.58, *p* < 0.001). Significant inverse correlations were observed between PSQI and LCQ scores at both baseline and follow-up. In multivariable analysis, LCQ score was an independent predictor of sleep quality during treatment (β = −0.453, *p* < 0.001), whereas demographic and physiological parameters showed no significant independent associations. No significant differences were detected between nintedanib and pirfenidone in terms of PSQI or LCQ outcomes. **Conclusions:** Cough-related quality of life is independently associated with sleep quality in patients with IPF receiving antifibrotic therapy. These findings highlight cough burden as a key determinant of patient-centered outcomes beyond traditional physiological measures. Targeted assessment and management of cough may represent an important strategy to improve sleep quality and overall quality of life in this population.

## 1. Introduction

Idiopathic pulmonary fibrosis (IPF) is a chronic, progressive fibrosing interstitial lung disease of unknown etiology that predominantly affects older adults and is histopathologically characterized by a usual interstitial pneumonia (UIP) pattern [1]. It remains a major cause of morbidity and mortality worldwide [2]. The clinical trajectory of IPF is highly variable, reflecting considerable inter-individual heterogeneity [3]. Although antifibrotic therapies such as pirfenidone and nintedanib have been shown to slow disease progression and improve survival, optimal management also requires a comprehensive approach addressing both disease-related complications and comorbid conditions [4]. In addition, emerging therapeutic strategies, including phosphodiesterase 4B inhibitors currently under investigation, may further expand treatment options in the future [5].

Chronic cough is among the most frequent and burdensome symptoms in IPF and has a substantial negative impact on health-related quality of life [6,7]. Structural remodeling of the lung parenchyma, including fibrosis-induced distortion and traction bronchiectasis, may alter airway mechanics and contribute to heightened cough sensitivity. Several mechanisms have been proposed to explain this relationship. Mechanical stress on peripheral airways may enhance the responsiveness of rapidly adapting receptors, thereby lowering the cough threshold [8]. Furthermore, disruption of normal lung architecture may impair inhibitory neural pathways involved in regulating the cough reflex [9]. Repetitive coughing itself may also contribute to disease progression through mechanical stress, potentially activating profibrotic pathways such as transforming growth factor-beta 1 (TGF-β1) signaling [10]. This process may create a self-reinforcing cycle in which cough promotes further fibrosis and symptom persistence [11,12].

Objective measures of cough frequency have been shown to correlate with patient-reported outcomes, including the Visual Analog Scale, Leicester Cough Questionnaire (LCQ), and Cough Symptom Score [13]. Prospective studies have demonstrated that cough burden may influence disease progression and prognosis in IPF [6,14,15]. In addition to its impact on daily functioning, chronic cough may contribute to sleep disruption, chest discomfort, and reduced overall well-being [16].

Sleep disturbances are increasingly recognized as a significant yet often underappreciated component of IPF. Previous studies have documented impaired sleep quality and alterations in sleep architecture, including reduced sleep efficiency, decreased slow-wave and rapid eye movement sleep, and increased sleep fragmentation [17,18,19,20,21]. Sleep quality in IPF is influenced by a complex interplay of factors, including age, pulmonary function impairment, dyspnea severity, nocturnal hypoxemia, comorbidities such as obstructive sleep apnea and gastroesophageal reflux disease, as well as psychological and symptom-related factors such as chronic cough [22].

The relationship between cough and sleep disturbance appears to be bidirectional. Poor sleep quality may increase central sensitization and enhance cough reflex sensitivity, while persistent coughing may disrupt sleep continuity. Despite growing evidence regarding symptom burden in IPF, the association between cough-related quality of life and sleep quality in patients receiving antifibrotic therapy remains insufficiently defined. Therefore, this study aimed to evaluate the relationship between cough burden and sleep quality and to investigate the potential impact of antifibrotic treatment on these patient-reported outcomes. We hypothesized that cough-related quality of life would be independently associated with sleep quality in this population.

## 2. Materials and Methods

### 2.1. Study Design and Patient Population

This study was designed as a cross-sectional analytical study conducted at a tertiary care center in Türkiye between January 2019 and December 2024. Patients with a diagnosis of idiopathic pulmonary fibrosis (IPF) who were receiving antifibrotic therapy (nintedanib or pirfenidone) were consecutively recruited from the pulmonology outpatient clinic. The diagnosis of IPF was established in accordance with the international ATS/ERS/JRS/ALAT guidelines. All cases were reviewed through multidisciplinary discussion (MDD) involving experienced pulmonologists and radiologists. Diagnosis was primarily based on high-resolution computed tomography (HRCT) findings demonstrating usual interstitial pneumonia (UIP) or probable UIP patterns. In cases with non-definitive imaging findings, additional clinical and radiological assessments were performed. Surgical lung biopsy was not required in the study population. Alternative causes of fibrotic interstitial lung disease were systematically excluded. Connective tissue disease-associated interstitial lung disease (ILD) was ruled out through detailed clinical evaluation and serological testing, while hypersensitivity pneumonitis and other ILDs were excluded based on exposure history, radiological features, and clinical assessment.

**Figure 1:**
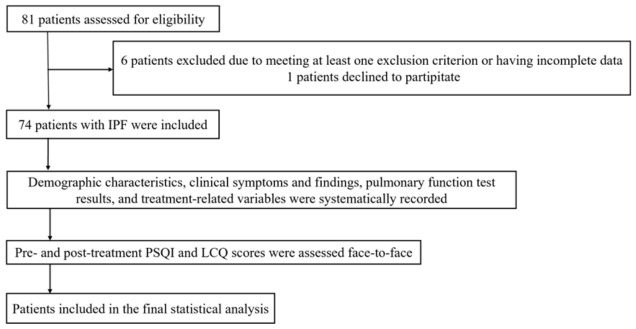
Study Flow Diagram.

The study was conducted in accordance with the Declaration of Helsinki and approved by the Mugla Sitki Kocman University Clinical Research Ethics Committee (protocol code: 240145; approval date: 28 August 2024). Written informed consent was obtained from all participants prior to enrollment. A total of 74 patients with idiopathic pulmonary fibrosis (IPF) who voluntarily agreed to participate and had accessible medical records were included in the study (Figure 1).

Inclusion criteria were: (1) age ≥ 18 years, (2) confirmed diagnosis of IPF according to international guideline criteria, and (3) ongoing antifibrotic treatment for a minimum of 3 months.

Exclusion criteria included: (1) prior diagnosis of obstructive sleep apnea, (2) high risk of sleep apnea defined as an Epworth Sleepiness Scale (ESS) score > 10 in combination with a STOP-Bang score ≥ 3, (3) presence of other interstitial lung diseases, (4) chronic airway diseases that could contribute to cough, (5) acute respiratory infection at the time of assessment, (6) acute exacerbation within the previous 3 months, (7) significant cognitive impairment affecting the reliability of questionnaire responses, and (8) incomplete clinical or questionnaire data.

### 2.2. Data Collection

Clinical and demographic characteristics—including age, sex, smoking exposure (pack-years), comorbidities, body mass index (BMI), pulmonary function parameters, and duration of antifibrotic therapy—were extracted from electronic medical records. Patient-reported outcomes were collected using validated questionnaires administered during routine outpatient visits.

Sleep quality was assessed using the Pittsburgh Sleep Quality Index (PSQI), and cough-related quality of life was evaluated using the Leicester Cough Questionnaire (LCQ). The Turkish versions of both instruments have been previously validated and demonstrated acceptable reliability, internal consistency, and construct validity in clinical settings [23,24].

All questionnaires were administered in a standardized manner through face-to-face interviews conducted by trained physicians. Follow-up assessments were performed during routine clinical visits while patients were receiving antifibrotic therapy. Baseline (pre-treatment) PSQI and LCQ data were not collected prospectively and were therefore obtained retrospectively based on patient recall during follow-up visits. This approach reflects routine clinical practice, where structured symptom assessments are not consistently performed at treatment initiation. Although structured interviews were used to enhance recall accuracy, the possibility of recall bias cannot be excluded and should be considered when interpreting baseline measurements.

Pulmonary function tests, including forced vital capacity (FVC), forced expiratory volume in one second (FEV1), and diffusing capacity for carbon monoxide (DLCO), were performed in accordance with established international guidelines.

### 2.3. Questionnaires and Assessments

#### 2.3.1. The Epworth Sleepiness Scale (ESS)

The ESS is a self-administered questionnaire with 8 questions that assesses daytime sleepiness. Dr Johns first developed the ESS for adults in 1990 and modified it in 1997 [25]. A score of 10 or higher is considered indicative of excessive sleepiness. The total score ranges from 0 to 24. Previous studies have reported ESS scores ranging from 2–10 in healthy control groups, 0–11 in individuals who snore, 4–23 in patients with obstructive sleep apnea syndrome, 0–6 in individuals with insomnia, and 2–16 in individuals with restless legs syndrome [26].

#### 2.3.2. STOP-Bang Questionnaire

The risk of obstructive sleep apnea (OSA) was assessed using the STOP-Bang questionnaire, a reliable, concise, and easy-to-use screening tool. The questionnaire consists of 8 dichotomous (yes/no) items assessing key clinical features of sleep apnea, yielding a total score of 0–8. Patients were classified according to OSA risk based on their total scores. A STOP-Bang score ≥ 3 demonstrates high sensitivity for detecting moderate-to-severe OSA (apnea–hypopnea index [AHI] > 15) and severe OSA (AHI > 30), with reported sensitivities of 93% and 100%, respectively. The corresponding negative predictive values are 90% and 100%. Scores between 0 and 2 indicate a low risk for moderate-to-severe OSA [27].

#### 2.3.3. Pittsburgh Sleep Quality Index (PSQI)

The PSQI was developed by Buysse et al. [28] as a self-administered questionnaire to assess sleep quality and sleep disturbances over the past month. It provides information on the type and severity of sleep disturbances. It consists of 24 items: 19 self-reported items completed by the individual and 5 items completed by a bed partner or roommate. Only the self-reported items are used to evaluate sleep quality; the items completed by the bed partner/roommate are not included in the scoring and serve solely to obtain additional clinical information. PSQI comprises seven components: subjective sleep quality, sleep latency, sleep duration, habitual sleep efficiency, sleep disturbances, use of sleep medication, and daytime dysfunction. Each component is scored on a 0–3 scale according to symptom frequency. The global score ranges from 0 to 21, with higher scores indicating poorer sleep quality and greater severity of sleep disturbance. A global score of ≥5 is considered clinically significant for sleep impairment.

#### 2.3.4. Leicester Cough Questionnaire (LCQ)

This instrument consists of 19 items assessing cough-related symptoms and their impact on quality of life, with each item scored on a 7-point Likert scale (1–7) based on the patient’s response. The scale includes three domains: physical (8 items), psychological (7 items), and social (4 items). Domain scores are calculated by dividing the sum of the item scores within each domain by the number of items in that domain, resulting in a score between 1 and 7 for each domain. The total score is calculated by summing the three domain scores, yielding a range of 3 to 21, with higher scores indicating better cough-related quality of life and lower scores reflecting greater cough burden [29]. Changes in LCQ scores have been interpreted based on established minimal clinically important difference (MCID) thresholds. A within-patient increase of approximately ≥1.3 points in the LCQ total score and ≥0.8, ≥0.9, and ≥0.8 points for physical, psychological, and social domain scores, respectively, is considered a clinically meaningful improvement following treatment [30].

### 2.4. Statistical Analyses

Statistical analyses were performed using IBM SPSS Statistics version 30.0 (IBM Corp., Armonk, NY, USA). Continuous variables were presented as mean ± standard deviation (SD) or median (interquartile range, IQR), as appropriate, based on distribution normality assessed using the Shapiro–Wilk test. Within-subject comparisons between baseline and follow-up measurements were performed using paired samples *t*-test for normally distributed variables and Wilcoxon signed-rank test for non-normally distributed variables. Between-group comparisons (nintedanib vs. pirfenidone) were conducted using independent samples *t*-test or Mann–Whitney U test, as appropriate. Correlations between variables were assessed using Spearman’s correlation analysis and presented in both graphical and tabular formats. Univariable linear regression analyses were first performed to identify factors associated with sleep quality. Variables with *p* < 0.10 in univariable analysis, along with clinically relevant variables, were included in the multivariable linear regression model. Multicollinearity was assessed using variance inflation factor (VIF), and no significant multicollinearity was detected. In the primary regression analysis, follow-up PSQI score was used as the dependent variable to reflect sleep quality during antifibrotic therapy. A *p*-value < 0.05 was considered statistically significant.

## 3. Results

Table 1 summarizes baseline demographic and clinical characteristics of the study population. At the time of initial diagnosis, the presenting symptoms were cough in 74.3% (n = 55), dyspnea in 59.4% (n = 44), sputum production in 17.5% (n = 13), and chest pain in 2.7% (n = 2).

All of the patients were receiving antifibrotic therapy for at least three months. Median anti-fibrotic treatment duration was 15 months (IQR: 7–48). Nintedanib was administered to 67.6% (n = 50) of the patients, whereas 32.4% (n = 24) received pirfenidone. The median daily dose of nintedanib was 300 mg (IQR: 300–300 mg), while the median daily dose of pirfenidone was 2400 mg (IQR: 2400–2400 mg). Long-term home oxygen concentrator use was present in 23.0% (n = 17) of the patients.

The comparison of clinical and functional parameters of the participants at baseline and during follow-up under antifibrotic treatment is presented in Table 2.

The median PSQI total score was 9 (IQR: 6–12) prior to antifibrotic treatment. According to the PSQI classification, 12.2% (n = 9) of patients had good sleep quality, whereas 87.8% (n = 65) had poor sleep quality at baseline. During antifibrotic therapy, the median PSQI total score decreased to 6 (IQR: 5–8), indicating improved overall sleep quality. A comparative analysis of PSQI subcomponent scores at baseline and during follow-up under antifibrotic treatment is presented in Table 3.

Assessment of the LCQ subdomains demonstrated lower scores prior to antifibrotic treatment, with significant improvements observed across all subdomains following therapy. Consistently, the total LCQ score increased during antifibrotic treatment compared with baseline. Detailed results are presented in Table 4.

**Figure 2:**
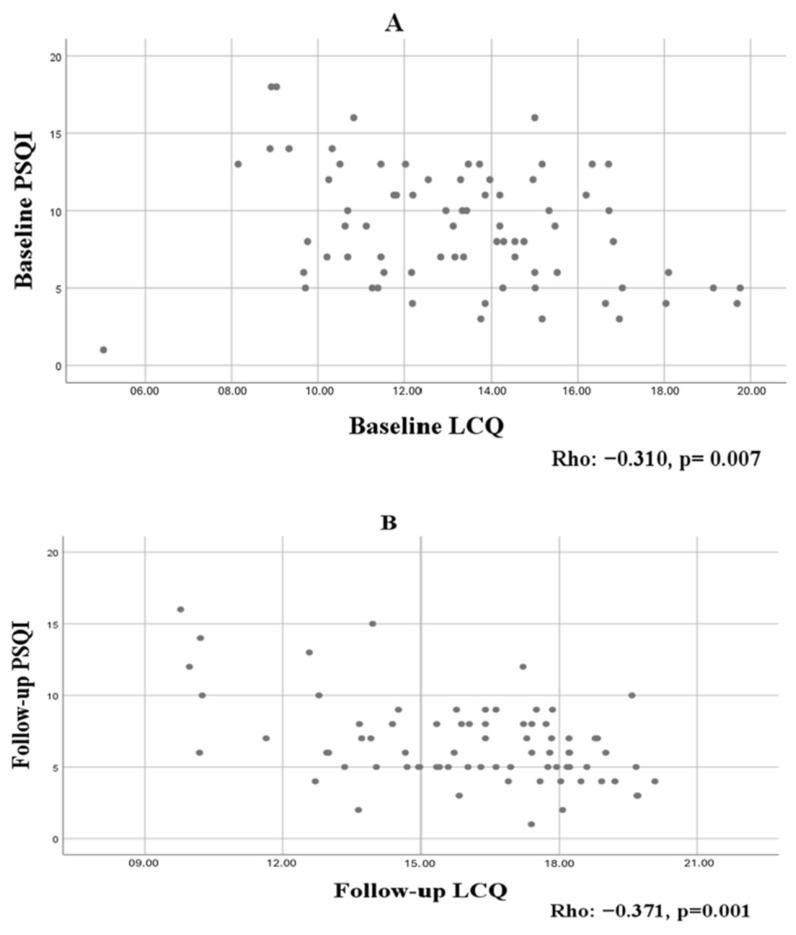
Scatter plots illustrating the correlation between total PSQI and LCQ scores at baseline (**A**) and follow-up (**B**).

A statistically significant inverse correlation was found between total PSQI and LCQ scores measured at baseline and during treatment (Figure 2)

No significant correlations were observed between PSQI and respiratory functional parameters at baseline. However, during treatment, PSQI demonstrated weak negative correlations with FVC (L) (rho = −0.248, *p* = 0.033) and FEV_1_ (L) (rho = −0.250, *p* = 0.032). LCQ showed a significant correlation only with baseline DLCO (%) (rho = 0.238, *p* = 0.041). During treatment, LCQ was significantly correlated with FVC (L) (rho = 0.343, *p* = 0.003) and FEV_1_ (L) (rho = 0.307, *p* = 0.008).

When the antifibrotic treatment groups were compared, no statistically significant differences were observed in PSQI and LCQ global scores measured during treatment between patients receiving nintedanib and those receiving pirfenidone (Table 5).

In univariable analysis, age and LCQ scores were associated with baseline PSQI scores. In multivariable analysis, LCQ total score remained an independent predictor of baseline sleep quality (Table 6). In contrast, smoking exposure (pack-years), comorbidity burden (CCI), body mass index (BMI), disease duration, and dyspnea severity (mMRC) were not independently associated with baseline PSQI (all *p* > 0.05). No evidence of multicollinearity was observed among the included variables (all variance inflation factor [VIF] values < 2).

Correlation analysis demonstrated that baseline PSQI was negatively associated with age (rho = −0.248, *p* = 0.017) and LCQ score (rho = −0.310, *p* = 0.007), indicating poorer sleep quality in older patients and in those with worse cough-related quality of life.

Similarly, in analyses of follow-up PSQI scores, LCQ total score was the only variable independently associated with sleep quality (Table 7). Other variables, including comorbidity burden (CCI), body mass index (BMI), age, dyspnea severity (mMRC), smoking exposure (pack-years), and treatment duration were not significantly associated with PSQI (all *p* > 0.05). No evidence of multicollinearity was observed among the included variables (all variance inflation factor [VIF] values < 2).

## 4. Discussion

The present study demonstrates a robust association between cough-related quality of life and sleep quality in patients with IPF undergoing antifibrotic therapy. Our findings indicate that cough burden is the primary independent determinant of sleep quality, underscoring the importance of symptom-based evaluation beyond conventional physiological parameters.

These results are consistent with previous studies suggesting that sleep impairment in IPF is multifactorial and not fully explained by pulmonary function indices alone. While objective physiological measures are essential for monitoring disease progression, they may not adequately reflect the subjective burden experienced by patients. Chronic cough, in particular, may disrupt sleep through recurrent nocturnal awakenings and fragmentation, thereby impairing restorative sleep.

Previous investigations have reported significantly impaired sleep quality in patients with IPF compared with healthy controls [22]. In these studies, sleep disturbance was not consistently associated with pulmonary function parameters but was closely linked to reduced health-related quality of life. Similarly, studies incorporating polysomnography and validated questionnaires have demonstrated that both nocturnal sleep quality and daytime functioning are adversely affected in IPF, with factors such as nocturnal hypoxemia and sleep fragmentation contributing to disease burden [21].

In our cohort, baseline sleep quality appeared to be influenced by multiple interacting factors, including age and cough-related quality of life. However, during antifibrotic therapy, cough-related quality of life emerged as the predominant determinant of sleep quality. This finding suggests that, as disease progression stabilizes under treatment, symptom burden—particularly cough—may become a more influential driver of patient-reported outcomes.

Although the duration of antifibrotic therapy varied considerably among patients, treatment duration was not independently associated with sleep quality in multivariable analysis. This observation indicates that improvements in sleep quality may be more closely related to changes in symptom burden rather than the duration of treatment exposure. Nevertheless, the wide range of treatment duration may still introduce heterogeneity and should be taken into account when interpreting the findings.

Consistent with previous reports, both antifibrotic agents demonstrated comparable effects on symptom-related outcomes, with no evidence of superiority between nintedanib and pirfenidone. These findings suggest that the observed improvements in cough-related quality of life and sleep quality may reflect a class effect rather than drug-specific differences.

Saunders et al. [6] reported a median LCQ score of 16.1 at the time of diagnosis, indicating that cough burden may be substantial even in patients with relatively preserved lung function. In that study, LCQ scores showed only weak yet statistically significant correlations with physiological measures such as FVC and DLCO, suggesting that cough-related quality of life may only partially reflect underlying disease severity. Longitudinal findings further indicated that cough burden tends to remain relatively stable in most patients, although worsening may occur in those with progressive disease. Importantly, LCQ was not identified as an independent predictor of mortality after adjustment for baseline lung function. In line with these findings, the association between cough-related quality of life and physiological parameters was limited in our cohort, supporting the notion that cough represents a distinct symptomatic dimension in IPF. This relative dissociation may be explained by mechanisms such as neural hypersensitivity and altered cough reflex pathways, which are not fully captured by conventional pulmonary function tests. Notably, during antifibrotic therapy, LCQ demonstrated significant correlations with FVC and FEV_1_ in our study. This observation suggests that, as disease progression becomes more stable under treatment, changes in cough-related quality of life may align more closely with functional parameters and may reflect treatment-related physiological dynamics rather than baseline disease severity alone.

In our cohort, cough and dyspnea were present in 74.3% and 59.4% of patients, respectively, consistent with previous reports identifying cough as a frequent and burdensome symptom in IPF [31]. LCQ total scores increased from 13.28 at baseline to 16.06 during follow-up, exceeding the minimal clinically important difference of 1.3 points [30], thereby indicating a clinically meaningful improvement. The most pronounced improvement was observed in the psychological domain, suggesting that treatment may have a substantial impact on the emotional burden associated with chronic cough.

Although smoking has been linked to disease progression in IPF [32], it was not significantly associated with either sleep quality or cough-related quality of life in our analysis. This finding implies that symptom-related factors may play a more direct role in shaping patient-reported outcomes than traditional risk factors.

Antifibrotic agents, including nintedanib and pirfenidone, are primarily aimed at slowing disease progression [33], while their effects on cough remain less clearly established. Some studies suggest that pirfenidone may reduce cough severity [11,34], whereas nintedanib may contribute to stabilization of symptom progression [35]. In our cohort, both treatments showed comparable effects on cough-related quality of life, indicating a similar potential for symptom modification.

Several limitations of this study should be considered. First, the cross-sectional design limits the ability to draw causal conclusions. Second, baseline PSQI and LCQ data were obtained retrospectively based on patient recall. Given the multidimensional structure of these questionnaires, this approach may introduce recall bias, as patients may not accurately remember their symptom severity prior to treatment initiation. As a result, baseline measurements may be subject to misclassification, and the magnitude of the observed changes in sleep quality and cough-related quality of life may have been either overestimated or underestimated.

Third, in the absence of a control group, the observed improvements in sleep quality and cough-related quality of life cannot be definitively attributed to antifibrotic therapy alone. Other factors that may have evolved during the follow-up period—such as the initiation of supplemental oxygen therapy, management of gastroesophageal reflux disease, or participation in pulmonary rehabilitation programs—could also have influenced these outcomes. Therefore, the results should be interpreted with caution.

In addition, objective assessments of sleep, such as polysomnography, were not performed, which limits the ability to comprehensively evaluate sleep architecture. Pulmonary function parameters were not included in the regression models to avoid potential multicollinearity. Despite these limitations, the study provides clinically meaningful insights into the role of symptom burden—particularly cough—in determining sleep quality in patients with IPF.

## 5. Conclusions

In conclusion, sleep quality remains impaired in patients with IPF despite ongoing antifibrotic therapy, although partial improvement may occur over time. Among the evaluated factors, cough-related quality of life emerged as the most important determinant of sleep quality, independent of conventional physiological parameters. These findings suggest that symptom burden—particularly chronic cough—represents a distinct and clinically relevant domain that is not fully captured by standard measures of disease severity.

Both antifibrotic agents demonstrated comparable effects on cough-related quality of life and sleep outcomes, indicating similar potential for symptom modulation. Importantly, the observed improvements in LCQ scores exceeded clinically meaningful thresholds, supporting the relevance of these changes from a patient-centered perspective.

Taken together, these results underscore the importance of incorporating routine assessment of cough and sleep disturbances into the clinical management of IPF. Addressing symptom burden may offer an additional opportunity to improve patient well-being beyond slowing disease progression.

Future prospective, multicenter studies with longitudinal follow-up and objective sleep assessments are warranted to further clarify these relationships and to evaluate the impact of targeted symptom management strategies on clinical outcomes.

## Figures and Tables

**Table 1:** Baseline Demographic and Clinical Characteristics of the Study Population.

Variable	Total (n = 74)
Age, years	68.6 ± 6.8
Male sex, n (%)	63 (85)
BMI, kg/m^2^	26.3 ± 3.4
Smoking status, n (%)	
Never	18 (24.3)
Former	47 (63.5)
Current	9 (12.2)
Pack-years, median (IQR)	25 (3.75–40)
CCI, median (IQR)	3 (2–3)
IPF duration, months, median (IQR)	15 (7–48)

BMI: Body mass index; CCI: Charlson comorbidity index; IPF: Idiopathic pulmonary fibrosis. Data are presented as mean ± standard deviation (SD) or median (interquartile range, IQR), as appropriate.

**Table 2:** Changes in Clinical and Pulmonary Function Parameters.

Parameter	Baseline	Follow-Up	*p* Value
FVC (%)	77.2 ± 17.8	75.3 ± 20.0	0.043 *
FVC (L)	2.67 ± 0.69	2.60 ± 0.68	0.201
FEV1 (%)	79.2 ± 17.4	75.8 ± 19.1	0.002 *
FEV1 (L)	2.26 ± 0.59	2.12 ± 0.60	0.004 *
FEV1/FVC	80.3 ± 7.2	80.4 ± 8.2	0.903
DLCO (%)	59.7 ± 23.7	58.2 ± 22.2	0.033 *
mMRC score	1.5 (1–2)	1.0 (1–2)	0.480

FVC: Forced vital capacity; FEV1: Forced expiratory volume in 1 s; DLCO: Diffusing capacity of the lung for carbon monoxide; mMRC: Modified Medical Research Council Dyspnea scale. Data are presented as mean ± standard deviation (SD) or median (interquartile range, IQR), as appropriate. *p* values were calculated using the paired *t*-test or Wilcoxon signed-rank test, as appropriate. * *p* < 0.05 indicates statistical significance.

**Table 3:** Within-Subject Changes in PSQI Total and Component Scores Following Antifibrotic Therapy.

Parameter	Baseline	Follow-Up	*p*-Value
Subjective sleep quality	1 (1–2)	1 (1–1)	<0.001 *
Sleep latency	2 (1–2)	1(1–1)	<0.001 *
Sleep duration	2 (1–3)	2 (1–2)	0.004
Habitual sleep efficiency	1.5 (1–3)	1 (0–2)	<0.001 *
Sleep disturbances	1 (1–2)	1 (1–1)	0.014
Use of sleeping medication	0 (0–0)	0 (0–0)	0.018
Daytime dysfunction	0 (0–1.25)	0 (0–0)	<0.001 *
PSQI total score	9 (6–12)	6 (5–8)	<0.001 *

PSQI: Pittsburgh Sleep Quality Index. Data are presented as median (interquartile range, IQR). Comparisons between baseline and follow-up values were performed using the Wilcoxon signed-rank test. * *p* < 0.05 indicates statistical significance.

**Table 4:** Changes in Leicester Cough Questionnaire (LCQ) Domain and Total Scores from Baseline to Follow-Up.

Domain	Baseline	Follow-Up	*p*-Value
Physical	4.75 ± 0.96	5.30 ± 0.90	<0.001 *
Psychological	3.76 ± 0.99	5.04 ± 1.05	<0.001 *
Social	4.73 ± 1.21	5.70 ± 0.98	<0.001 *
LCQ total score	13.28 ± 2.86	16.06 ± 2.58	<0.001 *

LCQ: Leicester Cough Questionnaire. Data are presented as mean ± standard deviation (SD). Comparisons between baseline and follow-up values were performed using the paired *t*-test. * *p* < 0.05 indicates statistical significance.

**Table 5:** Comparison of PSQI and LCQ Scores between Antifibrotic Treatment Groups during Follow-Up.

Parameter	Nintedanib (n = 50)	Pirfenidone (n = 24)	*p*-Value
PSQI total score	6 (5–8)	7 (5–8)	0.321
LCQ total score	16.11 ± 2.55	15.94 ± 2.71	0.785

PSQI: Pittsburgh Sleep Quality Index; LCQ: Leicester Cough Questionnaire. Data are presented as median (interquartile range, IQR) or mean ± standard deviation (SD), as appropriate. Comparisons between groups were performed using the Mann–Whitney U test or independent *t*-test, as appropriate.

**Table 6:** Univariable and Multivariable Linear Regression Analysis of Factors Associated with Baseline PSQI Score.

Variable	Univariable B (95% CI)	*p*-Value	Multivariable B (95% CI)	*p*-Value
CCI	0.323 (−0.508, 1.153)	0.441	0.629 (−0.212, 1.469)	0.140
BMI	0.175 (−0.087, 0.437)	0.188	0.112 (−0.144, 0.368)	0.385
Age	−0.138 (−0.265, −0.011)	0.033	−0.153 (−0.286, −0.020	0.024
mMRC	−0.641 (−1.891, 0.609)	0.310	−0.795 (−1.979, 0.389)	0.185
Pack-years	−0.004 (−0.051, 0.043)	0.867	−0.001 (−0.046, 0.044)	0.972
LCQ total score, baseline	−0.386 (−0.687, −0.086)	0.012	−0.348 (−0.646, −0.049)	0.023

CCI: Charlson Comorbidity Index; BMI: Body mass index; mMRC: Modified Medical Research Council (Dyspnea scale); LCQ: Leicester Cough Questionnaire. B values represent unstandardized regression coefficients. Variables with *p* < 0.10 in univariable analysis and clinically relevant variables were included in the multivariable model.

**Table 7:** Univariable and Multivariable Linear Regression Analysis of Factors Associated with Follow-Up PSQI Score.

Variable	Univariable B (95% CI)	*p*-Value	Multivariable B (95% CI)	*p*-Value
CCI	0.072 (−0.012, 0.157)	0.092	0.472 (−0.140, 1.085)	0.116
BMI	0.018 (−0.009, 0.045)	0.196	−0.110 (−0.290, 0.070)	0.254
Age	0.002 (−0.011, 0.016)	0.762	−0.082 (−0.177, 0.013)	0.091
mMRC	0.046 (−0.058, 0.149)	0.381	−0.280 (−0.978, 0.418)	0.437
Pack-years	0.001 (−0.004, 0.006)	0.630	0.028 (−0.005, 0.060)	0.099
LCQ total score, follow-up	−0.044 (−0.078, −0.009)	0.013	−0.544 (−0.795, −0.293)	<0.001
Treatment duration, months	0.004 (−0.027, 0.035)	0.816	−0.004 (−0.032, 0.024)	0.785

CCI: Charlson Comorbidity Index; BMI: Body mass index; mMRC: Modified Medical Research Council (Dyspnea scale); LCQ: Leicester Cough Questionnaire. B values represent unstandardized regression coefficients. Variables with *p* < 0.10 in univariable analysis and clinically relevant variables were included in the multivariable model. Treatment duration was included to account for variability in treatment exposure.

## Data Availability

The data supporting the findings of this study are available from the corresponding author upon reasonable request.
